# Perceived Burdensomeness, Thwarted Belongingness and Fearlessness About Death in Italian university students: validation of the INQ-15 and the ACSS-FAD

**DOI:** 10.1192/j.eurpsy.2022.645

**Published:** 2022-09-01

**Authors:** S. Magliocca, D. Romano, F. Madeddu, P. Zeppegno, C.M. Gramaglia, R. Calati

**Affiliations:** 1 University of Milan-Bicocca, Department Of Psychology, Milan, Italy; 2 University of Salento, Department Of History, Society And Human Studies, Lecce, Italy; 3 Azienda Ospedaliero Universitaria Maggiore della Carità, S.c. Psichiatria, Novara, Italy; 4 Università del Piemonte Orientale, Medicina Traslazionale, Novara, Italy; 5 Nîmes University Hospital, Department Of Adult Psychiatry, Nîmes, France

**Keywords:** Perceived burdensomeness, Thwarted belongingness, Fearlessness About Death, Suicide

## Abstract

**Introduction:**

The *Interpersonal Needs Questionnaire* (INQ-15) and the *Acquired Capability for Suicide Scale - Fearlessness About Death* (ACSS-FAD) have been introduced to evaluate the theoretical constructs posit by Joiner’s Interpersonal Psychological Theory of Suicide (IPTS).

**Objectives:**

The present study aimed to evaluate the psychometric properties of the INQ-15 (which measures Thwarted Belongingness, TB, and Perceived Burdensomeness, PB) and the ACSS-FAD (measurement of Fearlessness About Death, FAD, dimension of the acquired capability) in a population of Italian university students.

**Methods:**

Since there was no Italian version of the ACSS-FAD, we have translated it through an accurate multistage procedure. ACSS-FAD and INQ-15 have been administered to a sample of 1,665 Italian university students. We analyzed the factorial structure of the INQ-15 and the ACSS-FAD, their reliability, criterion, convergent and discriminant validity.

**Results:**

Principal Component Analysis confirmed a two-dimensional structure for INQ-15 and a one-factor structure for ACSS-FAD. Internal consistency reliability of the scales was good, respectively TB: α = .85; PB: α = .90; and FAD: α = .85. The INQ-15 demonstrated concurrent associations with suicidal ideation, while the ACSS-FAD with a history of suicidal planning/suicide attempt. Convergent and discriminant validity were also in line with previous studies.

**Conclusions:**

Both INQ-15 and ACSS-FAD appropriately capture the respective constructs, proving to be valid measures for the assessment of suicide risk factors among Italian university students according to the IPTS. The valuable psychometric properties of the two scales established with this study in the Italian context encourages their use to advance the clinical understanding and prevention of suicide.

**Disclosure:**

No significant relationships.

